# Editorial

**Published:** 1900-10-15

**Authors:** 


### EDITORIAL.

A NEW dental college has been launched in one of the Pacific coast cities, and announces that it has made arrangements with the Educational Department of the Young Men's Christian Association to take charge of the preliminary educational requirements of its students. It announces that night sessions will be held and all students will be required to attend the classes, and that the work will be done so as to conform to the requirements of the

National Association of Dental Faculties. We would not cast any reflections on any of the work done by the Young Men's Christian Association. But we seriously doubt the ability of such organization to provide that mental training which is essential to one who intends to qualify himself for the practice of a profession, necessitating the mastery of so many of the broad sciences and in so short a time as does our profession. Students so prepared will greatly embarrass the best teachers. The incapacitated student is liable to become so demoralized that he looses moral restraint and resorts to trickery to pass his examinations, and when graduated he is so handicapped that he can not compete with other members of his profession and soon drifts into quackery.

---

We have, during the past year, received many circulars through the mail soliciting prosthetic work to be done in specially equipped laboratories at what seems to us starvation prices for the men who do the work. We have not had the courage to test any of these remarkable offers, and have no reason to believe they are not genuine; but we should like to know how they manage to pay rent or the printer and buy postage stamps. We suppose there is a demand for such services, or they would not be so much in evidence. And if this is a fact, what does it signify? Is the practice of prosthetic dentistry degenerating from an art to a kind of "hand-me-down" traffic? There are some signs which would lead us to think that dental prosthesis has not much future as an artistic or scientific accomplishment. Students in colleges do not like it, consequently they will not make themselves proficient. If compelled to practice it after graduation it is from the bread and butter necessity standpoint, hence being perfunctory it is very often unsatisfactory to the patient and demoralizing to the dentist. The very work itself is degraded until it naturally finds its place as a commodity, to be had for prices which will not justify a professional man engaging in this kind of work. If this is to be the future of prosthetic dentistry, is it not time we were recognizing the fact and taking some measures to stem the tide? What these measures shall be ought to be discovered very quickly. Naturally we look first to the college where our dentists are educated. Prosthetic technics and lectures on the art and science of this important branch must receive more attention. New methods, new facilities, and, may be, new teachers, should be given careful consideration. Let us give prosthetic art as dignified and important a place in our educational system as any other branch of the curriculum.

